# Current News

**Published:** 1896-07

**Authors:** 


### Current News.

### NATIONAL ASSOCIATION OF DENTAL EXAMINERS.

The Twelfth Annual Session will be held at Saratoga Springs, N. Y., commencing at ten o'clock A.M., Monday, August 3, and will continue in session during the proceedings of the American Dental Association. It is earnestly requested that all State and Territorial Boards of Dental Examiners will send delegates.

CHARLES A. MEEKER,
*Secretary and Treasurer.*

No. 29 FULTON STREET, NEWARK, N. J.

### NEW JERSEY STATE DENTAL SOCIETY.

The Twenty-sixth Annual Session of the New Jersey State Dental Society will be held in the Auditorium, at Asbury Park, N. J., July 29, 30, and 31. Essays on subjects pertaining to dentistry will be read by the most eminent men in the profession. Twenty-seven clinics on the afternoon of the 29th will be of the utmost interest. Fifteen hundred feet of space for exhibitions already nearly filled. Many new electrical appliances for the first time exhibited. New Jersey head-quarters, the Columbia; rates, $2.50 to $3 per day. Board in the Park from $8 to $45 per week. Cut off the time and come and see us.

CHARLES A. MEEKER, D.D.S.,
*Secretary.*

NEWARK, N. J.

### ILLINOIS STATE DENTAL SOCIETY.

The Thirty-second Annual Meeting of the Illinois State Dental Society was held at Springfield, May 12 to 15, 1896. A good programme was carried out, and a large attendance was present. The following officers were elected: President, C. R. Taylor, Streator;

Vice-President, E. B. David, Aledo; Secretary, Louis Ottofy, Chicago; Treasurer, E. D. Swain, Chicago; Librarian, J. R. Rayburn, Fairbury. The next meeting will be held at Peoria, beginning on the second Tuesday in May, 1897.

LOUIS OTTOFY,
*Secretary.*

MASONIC TEMPLE, CHICAGO.

---

### CINCINNATI ACADEMY OF DENTISTRY.

AT the regular monthly meeting of the Cincinnati Academy of Dentistry, held Monday evening, April 27, 1896, the following officers were elected for the ensuing year,—viz.: President, W. T. McLean, M.D., D.D.S.; Vice-President, A. I. F. Buxbaum, M.D., D.D.S.; Treasurer, J. F. Clayton, D.D.S.; Secretary, Wm. Lockman, Jr., D.D.S.

